# Sex‐based differences in early and late uveal melanoma‐related mortality

**DOI:** 10.1002/cam4.5458

**Published:** 2022-11-18

**Authors:** Gustav Stålhammar

**Affiliations:** ^1^ Division of Eye and Vision, Department of Clinical Neuroscience St. Erik Eye Hospital, Karolinska Institutet Stockholm Sweden; ^2^ St. Erik Eye Hospital Stockholm Sweden

**Keywords:** choroidal melanoma, conditional, gender, men, metastasis, prognosis, sex, survival, uveal melanoma, women

## Abstract

**Background:**

It is debated if there are sex‐based differences in survival for patients with uveal melanoma. Previous observations of higher mortality for men in studies with <10‐year follow‐up have not been replicated in studies with longer follow‐up. It is therefore hypothesized that women have a worse survival in later periods.

**Methods:**

All patients diagnosed with primary uveal melanoma in Sweden between 1980 and 2017 were included (*n* = 2032). Survival differences between men and women in early (<10 years from diagnosis) and late (≥10 years) periods were analyzed.

**Results:**

At baseline, there were no significant differences in mean patient age, tumor thickness, diameter, ciliary body involvement, primary treatment modality, or in American Joint Committee on Cancer (AJCC) T‐category between men and women. In total, 764 patients (425 women and 339 men) survived and were followed ≥10 years. In this group, men were significantly younger, but there were no differences in baseline tumor thickness, diameter, ciliary body involvement, primary treatment, or AJCC T‐category. In competing risk analysis, women had higher incidence of uveal melanoma‐related mortality in the late period (*p* = 0.036). In univariate Cox regression, male (HR 1.2, *p* = 0.049) and female sex (HR 1.8, *p* = 0.034) were significant predictors of uveal melanoma‐related mortality in the early and late periods, respectively.

**Conclusion:**

Women with uveal melanoma have better survival in the first decade after diagnosis. Thereafter, female survivors are significantly older than men and have a higher incidence of uveal melanoma‐related mortality.

## Graphical abstract

In this nationwide Swedish retrospective survey of 2032 patients, women had lower hazard for uveal melanoma‐related mortality in the first decade after diagnosis, but higher hazard thereafter.

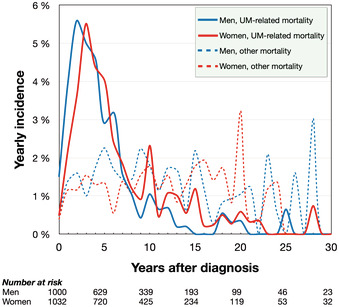

## 1 INTRODUCTION

At the time of uveal melanoma (UM) diagnosis, about 2% of patients have radiologically detectable metastases.^1^ Fifteen years after diagnosis, however, the relative survival for UM patients is only 60%.^2^ Once metastases have been detected, no effective treatment alternatives are available and the median patient survival is about 1 year.^3^, ^4^ Response rates to immune checkpoint inhibitors remain low, but other pathways may be targetable.^5^ Recently, encouraging results were presented, when treatment with tebentafusp was shown to prolong median overall survival to 22 months in a group of previously untreated HLA‐ A*02:01–positive patients with metastatic disease.^6^

Clinical factors associated with a poor prognosis include older age at presentation, greater tumor diameter and apical thickness, ciliary body involvement, extraocular extension, and more advanced American Joint Committee on Cancer (AJCC) T‐category and stage.^7^, ^8^, ^9^ The importance of sex for UM prognosis has been debated. Overall, the incidence is similar in men and women.^10^, ^11^, ^12^ Some observations indicate an increased age‐adjusted incidence in men.^13^, ^14^, ^15^ Male sex has also been identified as an independent predictor of metastasis and UM‐related death within the first decade after diagnosis.^16^, ^17^, ^18^, ^19^ Notably, other large randomized studies or retrospective cohort studies with longer follow‐up have failed to identify sex‐based differences in survival rates.^20^, ^21^, ^22^, ^23^ Some observations even indicate that young women have a relatively worse prognosis than young men.^24^

In this nation‐wide study, survival between men and women in the first decade after UM diagnosis is compared, as well as in the period after the first decade. Based on the previous reports with long follow‐up that have not been able to replicate the worse survival for men that has been found in studies with shorter follow‐up, it is hypothesized that men indeed have a worse survival in the first decade whereas the situation may be reversed in the later period so that their long‐term prognosis converge. This would have consequences for our current understanding of this disease, for how we inform patients and relatives about the prognosis and could have impact on future treatment studies.

## 2 METHODS

### 2.1 Patient selection

All patients that were diagnosed with primary UM in Sweden between January 1, 1980, and December 31, 2017, and had data available in the national digitalized UM treatment registry were included in the study (*n* = 2032). The registry is continuously updated with dates and causes of death from the national Cause of Death Registry and has previously been estimate to capture more than 95% of UM patients.^25^ The Cause of Death Registry reports the underlying cause of death, according to the International Classification of Diseases (ICD) code in use at the time of death. The method of establishing the cause of death is coded as autopsy, clinical examination or forensic investigation, along with a more specified subgrouping of the clinical examination.^26^ To reduce the number of classification errors, for example, death from metastatic UM coded as death from metastatic cutaneous melanoma, the registry had been cross‐checked against other diagnoses in the national Cancer Registry and against hospital medical records, as described previously.^27^, ^28^, ^29^

The study follows the tenets of the Declaration of Helsinki and the research group's internal data security policy for sensitive data. Ethical permission was obtained from the Swedish Ethical Review Authority (record number 2020–02835). According to the approved ethics application, the requirement for written informed consent was waived as this was a retrospective study that did not require collection of identifiable health information including patient names, personal identifiers, addresses, other contact details, or photographs, that did not involve sampling or analysis of biological samples, and did not affect treatment or follow‐up of the patients. The raw data used for this study are available as a supplementary file.

### 2.2 Statistical methods

*p* values below 0.05 were considered statistically significant, all *p* values being two‐sided. For comparisons of continuous variables, Student's *t*‐tests were used as no variable deviated significantly from normal distribution (Shapiro–Wilk test *p* > 0.05). In comparisons of categorical variables, I used two‐by‐two contingency tables and Pearson chi‐square (χ^2^) tests (if all fields had a sample of >5) or Fisher's exact tests (if any field had a sample of <5). Tumor volumes were estimated assuming a semi ellipsoid shape:^30^

$$\text{Volume of tumor}=\frac{\pi }{6}\times t\times {\mathrm{lbd}}^{2}$$

The cumulative incidence of UM‐related mortality was plotted in cumulative incidence function estimates from competing risks data with the cmprsk package for R, and the equality of survival distributions was tested with Gray's test for equality.^31^ Overall survival for men and women were plotted in Kaplan–Meier curves and the log‐rank test applied. Considering the primary hypothesis (men have worse survival in the first decade after diagnosis, whereas the situation may be reversed in the later period) crossing survival curves were expected. In such situations, there is clear departure from proportional hazards and the log‐rank test is inappropriate. Therefore, it was determined beforehand that in the event of crossing overall survival curves the partial slopes rank‐sum test would be used, which has been shown to be more reliable in this situation (calculated with Online Application for Survival Analysis 2, at https://sbi.postech.ac.kr/oasis2/, Structural Bioinformatics Lab, Pohang University of Science and Technology, South Korea).^32^, ^33^ The predetermined cutoff for the early and late period was 10 years after diagnosis, based on previous observations of UM‐related mortality curves for women overtaking men at approximately this point in time.^21^, ^23^, ^34^ This phenomenon was not commented in the original publications. When calculating survival differences in the first decade after treatment, patients with ≥10 years of event‐free follow‐up as well as patients that suffered from any event (UM‐related death or death from any other cause) after ≥10 years were coded as event‐free and censored at 10 years. When calculating survival differences after the first decade, patients that suffered from any event within 10 years or had been followed for less than 10 years were removed from analysis. This operation imitates Dogrusöz et al., who examined prognostic factors 5 years after enucleation for UM.^34^ For comparisons of association with UM‐related death, multivariate Cox regression hazard ratios (HR) were calculated. All statistical analyses except crossing survival distributions and cumulative incidence function estimates were performed using IBM SPSS statistics version 27 (Armonk, NY, USA) and GraphPad Prism version 9.3.0 (San Diego, CA, USA).

## 3 RESULTS

### 3.1 Descriptive statistics

Of the 2032 included patients, 1,000 (49%) were men and 1,032 (51%) were women. No patient was classified as having another sex. Their mean age at diagnosis was 63.2 years (SD 13.7) and their tumors had a mean largest basal diameter of 11.0 mm (SD 3.9) and a mean thickness of 5.8 mm (3.0). Twelve hundred and thirty‐five patients underwent plaque brachytherapy with ruthenium‐106 as primary treatment, 269 underwent plaque brachytherapy with iodine‐125, and 528 patients underwent enucleation. Nine hundred and ninety‐nine patients had deceased before last follow‐up. Five hundred and forty‐six of those (55%) had deceased from metastatic UM. The median follow‐up for the 1,041 survivors was 10.5 years (IQR 11.1, Table 1).

**TABLE 1:** Demographics and clinical features of study patients and tumors

*n*	2032
Sex, *n* (%)	
Men	1000 (49)
Women	1032 (51)
Ciliary body involvement, *n* (%)	
No	1969 (97)
Yes	63 (3)
Mean age at diagnosis, years (SD)	63.2 (13.7)
Mean tumor thickness, mm (SD)	5.8 (3.0)
Mean tumor diameter, mm (SD)	11.0 (3.9)
Primary treatment, *n* (%)	
^106^Ru brachytherapy	1235 (61)
^125^I brachytherapy	269 (13)
Enucleation	528 (26)
AJCC T‐category, *n* (%)	
1a	603 (30)
1b	5 (<1)
1c + 1d	0 (0)
2a	686 (34)
2b	4 (<1)
2c	1 (<1)
2d	0 (0)
3a	378 (19)
3b	6 (<1)
3c + 3d	0 (0)
4a	92 (5)
4b	2 (<1)
4c + 4d	0 (0)
N/a	255 (13)
Follow‐up years, median^a^ (IQR)	10.5 (11.1)

Abbreviations: ^106^Ru, Ruthenium‐106 plaque; ^125^I, Iodine‐125 plaque; AJCC, American Joint Committee on Cancer; IQR, interquartile range; SD, standard deviation.

^a^ For survivors.

**FIGURE 1:**
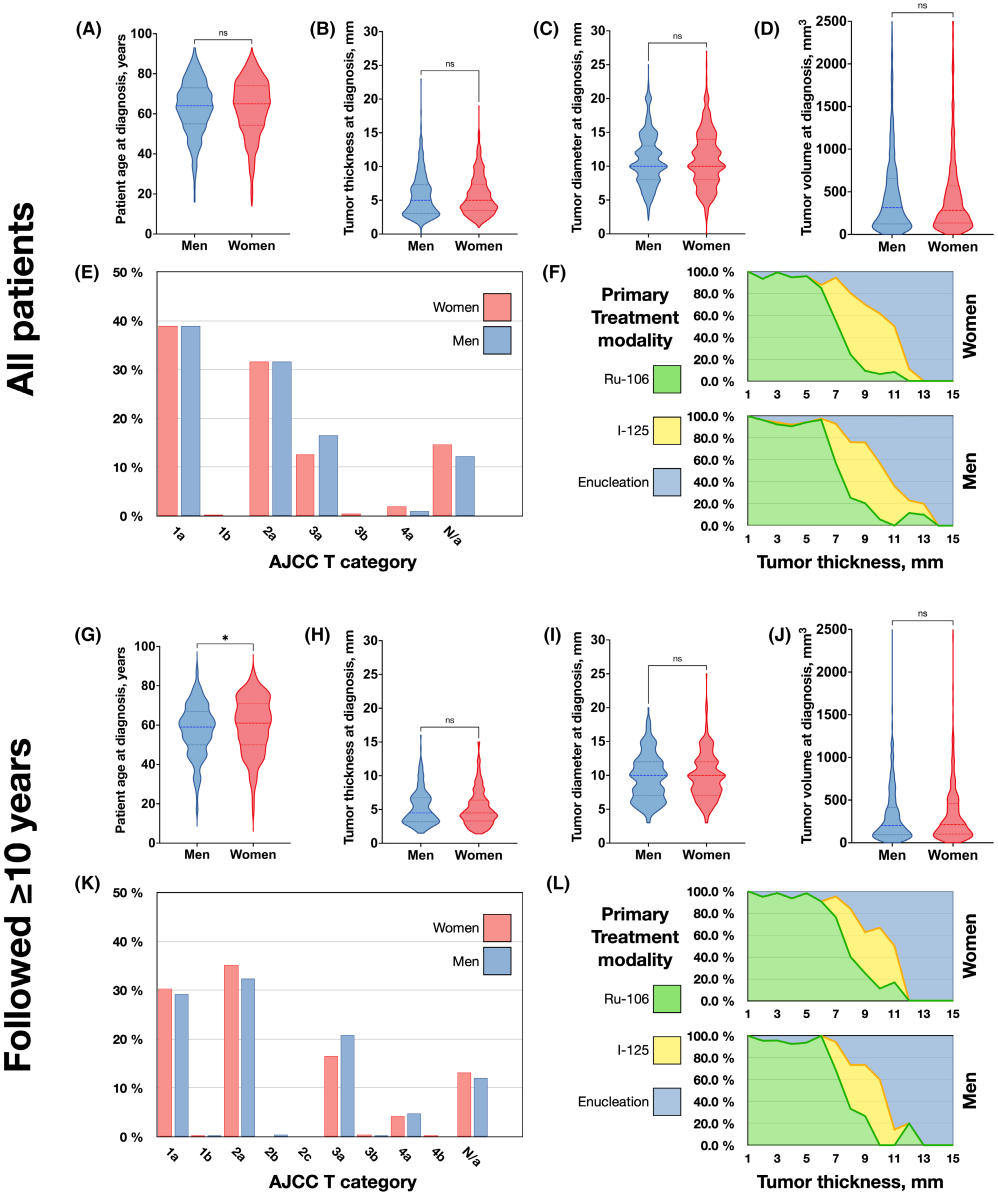
Patient, tumor, and treatment characteristics for all patients (top) and patients followed ≥10 years (bottom). At baseline, there were no significant differences in (A) patient age, (B) tumor apical thickness, (C) tumor diameter, or (D) tumor volume. (E) Distribution of American Joint Committee on Cancer (AJCC) T category between all men and women. (F) Primary treatment modality for all women (top) and men (bottom) across tumor apical thickness. Among patients followed ≥10 years, (G) men were significantly younger (*p* = 0.02), but there were no significant differences in baseline (H) tumor apical thickness, (I) tumor diameter, or (J) tumor volume. (K) Distribution of AJCC T category between men and women followed ≥10 years. (L) Primary treatment modality for women (top) and men (bottom) followed ≥10 years across tumor apical thickness. *p < 0.05. ns, non‐significant. Ru‐106, Ruthenium‐106 plaque brachytherapy. I‐125. Iodine‐125 plaque brachytherapy.

There were no significant differences in mean age at diagnosis, tumor thickness, diameter, ciliary body involvement, primary treatment or AJCC T‐category between men and women at the time of diagnosis (Table 2, Figure 1A–F).

**TABLE 2:** Patient, tumor, and treatment features of women versus men

	Women, *n* = 1032	Men, *n* = 1000	*p*
Mean age at diagnosis, years (SD)	63.3 (14.1)	63.0 (13.4)	0.62
Mean tumor thickness, mm (SD)	5.7 (2.9)	5.9 (3.1)	0.22
Mean tumor diameter, mm (SD)	11.0 (2.9)	11.1 (4.1)	0.56
Ciliary body involvement, *n* (%)			
No	1006 (97)	963 (96)	0.12
Yes	26 (3)	37 (4)	
Primary treatment, *n* (%)			
^106^Ru brachytherapy	632 (61)	603 (60)	0.87
^125^I brachytherapy	133 (13)	136 (14)	
Enucleation	267 (26)	261 (26)	
AJCC T‐category, *n* (%)			
1a	312 (30)	291 (29)	0.12
1b	3 (<1)	2 (<1)	
1c + 1d	0 (0)	0 (0)	
2a	362 (35)	324 (32)	
2b	0 (0)	4 (<1)	
2c	0 (0)	1 (<1)	
2d	0 (0)	0 (0)	
3a	170 (16)	208 (21)	
3b	4 (<1)	2 (<1)	
3c + 3d	0 (0)	0 (0)	
4a	44 (4)	48 (5)	
4b	2 (<1)	0 (0)	
4c + 4d	0 (0)	0 (0)	
N/a	135 (13)	120 (12)	

Abbreviations: N/a, not available; SD, standard deviation.

### 3.2 Patients followed less than 10 years

In the first decade after diagnosis, 779 patients suffered from a UM‐related death (*n* = 496) or other death (*n* = 283). Four hundred and eighty‐nine patients were followed <10 years. Considering that patients with ≥10 years of event‐free follow‐up as well as patients that suffered from any death after ≥10 years were coded as event‐free and censored at 10 years in analysis of survival in the first decade after diagnosis, their baseline characteristics were identical as in the full cohort (Table 2).

Of the 496 patients that died from UM, 239 (48%) were women and 257 (52%) were men. There were no significant sex differences in the distribution of ciliary body involvement or in age at diagnosis, tumor thickness, tumor diameter, or in treatment (Table 3).

**TABLE 3:** Patient, tumor, and treatment features of women versus men followed <10 years*

	Women, *n* = 365	Men, *n* = 414	*p*
Dead from any cause, *n* = 779			
Mean age at diagnosis, years (SD)	67.4 (12.5)	67.4 (12.3)	1.0
Mean tumor thickness, mm (SD)	6.5 (3.0)	6.7 (3.5)	0.45
Mean tumor diameter, mm (SD)	12.5 (4.0)	12.7 (4.3)	0.72
Ciliary body involvement, *n* (%)			
No	352 (96)	385 (93)	0.10
Yes	13 (4)	26 (7)	
Primary treatment, *n* (%)			
^106^Ru brachytherapy	180 (49)	202 (49)	0.98
^125^I brachytherapy	41 (11)	48 (12)	
Enucleation	144 (39)	164 (40)	

	Women, *n* = 239	Men, *n* = 257	*p*
Dead from uveal melanoma, *n* = 496			
Mean age at diagnosis, years (SD)	64.4 (12.4)	63.8 (11.7)	0.58
Mean tumor thickness, mm (SD)	7.0 (3.0)	7.2 (3.5)	0.44
Mean tumor diameter, mm (SD)	13.1 (4.0)	13.6 (4.2)	0.24
Ciliary body involvement, *n* (%)			
No	229 (96)	236 (92)	0.09
Yes	10 (4)	21 (8)	
Primary treatment, *n* (%)			
^106^Ru brachytherapy	109 (46)	113 (44)	0.63
^125^I brachytherapy	34 (14)	31 (12)	
Enucleation	96 (30)	113 (44)	

a Patients with ≥10 years of event‐free follow‐up as well as patients that suffered from any event (uveal melanoma‐related death or death from any other cause) after ≥10 years were coded as event‐free and censored at 10 years.

Of the 283 patients that deceased from other causes, 126 (45%) were women and 157 (56%) were men, and there were no significant differences in their age at diagnosis (Student's *t*‐test *p* = 0.88), tumor thickness (*p* = 0.27), diameter (*p* = 0.86), ciliary body involvement (χ^2^ *p* = 0.49), or AJCC T‐category (*p* = 0.85).

Of the 489 patients that were followed for less than 10 years, 243 (50%) were women and 246 (50%) were men, and there were no significant differences in their age at diagnosis (Student's *t*‐test *p* = 0.56), tumor thickness (*p* = 0.87), diameter (*p* = 0.66), ciliary body involvement (χ^2^ *p* = 0.72), or AJCC T‐category (*p* = 0.75).

### 3.3 Patients followed for more than 10 years

A total of 764 patients (425 women and 339 men) were followed for more than 10 years. In this group, men were significantly younger (57.5 vs. 59.6 years at diagnosis, corresponding to 67.5 vs. 69.5 years a decade later, *p* = 0.02) but there were no significant differences in tumor thickness, diameter, ciliary body involvement, primary treatment, or AJCC T‐category at the time of diagnosis (Table 4, Figure 1G–L).

**TABLE 4:** Patient, tumor, and treatment features of women versus men followed ≥10 years

	Women, *n* = 425	Men, *n* = 339	*p*
Mean age at diagnosis, years (SD)	59.6 (14.3)	57.5 (13.1)	0.04
Mean tumor thickness, mm (SD)	5.2 (2.6)	5.3 (2.6)	0.69
Mean tumor diameter, mm (SD)	9.9 (3.3)	9.7 (3.4)	0.54
Ciliary body involvement, *n* (%)			
No	414 (97)	332 (96)	0.64
Yes	11 (3)	7 (4)	
Primary treatment, *n* (%)			
^106^Ru brachytherapy	294 (69)	233 (69)	0.56
^125^I brachytherapy	28 (7)	29 (9)	
Enucleation	103 (24)	77 (23)	
AJCC T‐category, *n* (%)			
1a	165 (39)	132 (39)	0.12
1b	1 (<1)	0 (0)	
1c + 1d	0 (0)	0 (0)	
2a	134 (32)	107 (32)	
2b	0 (0)	0 (0)	
2c	0 (0)	0 (0)	
2d	0 (0)	0 (0)	
3a	53 (12)	56 (17)	
3b	2 (<1)	0 (0)	
3c + 3d	0 (0)	0 (0)	
4a	8 (2)	3 (1)	
4b	0 (0)	0 (0)	
4c + 4d	0 (0)	0 (0)	
N/a	62 (15)	41 (12)	

Abbreviations: N/a, not available; SD, standard deviation.

### 3.4 Cumulative incidence of UM‐related mortality and overall survival

**FIGURE 2:**
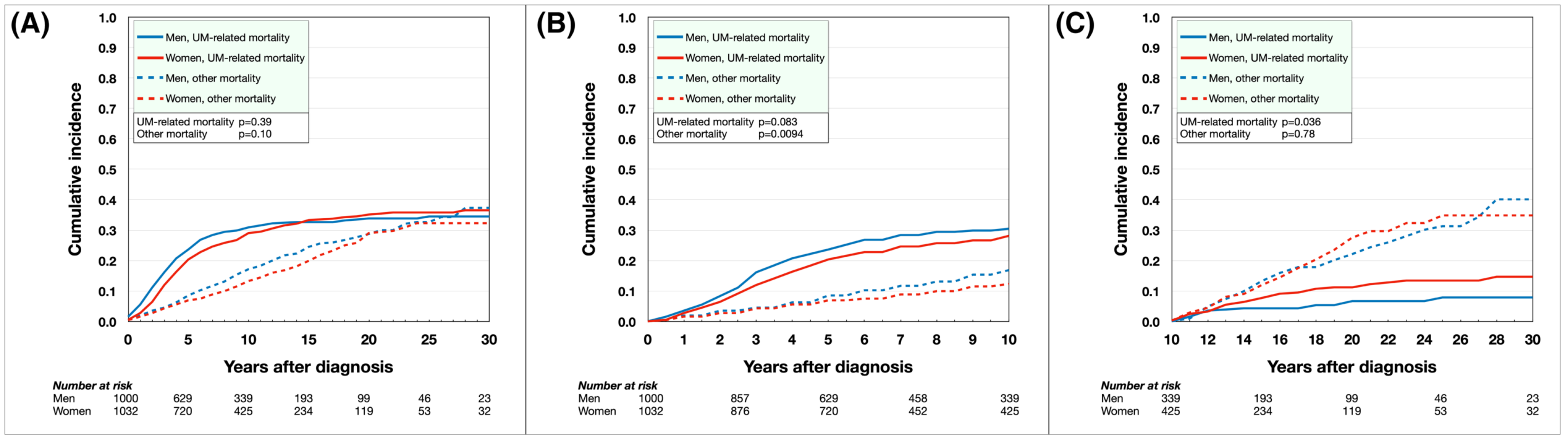
Cumulative incidence of UM‐related mortality in competing risk analysis. (A) During the period from 0 to 30 years after diagnosis, women initially had lower incidence of UM‐related mortality, but the curves crossed 14 years after diagnosis, after which men had lower incidence of UM‐related mortality. Overall, the incidence of UM‐related mortality was equal between men and women (Gray's test for equality *p* = 0.39) Similarly, there was no significant difference in incidence of death from other causes (*p* = 0.10). (B) In the first decade after diagnosis, the incidence of UM‐related mortality was equal between men and women (*p* = 0.083). Men had greater incidence of death from other causes (*p* = 0.0094). (C) After the first decade, women had significantly higher incidence of UM‐related mortality (*p* = 0.036), but there was no significant difference in death from other causes (*p* = 0.78).

**FIGURE 3:**
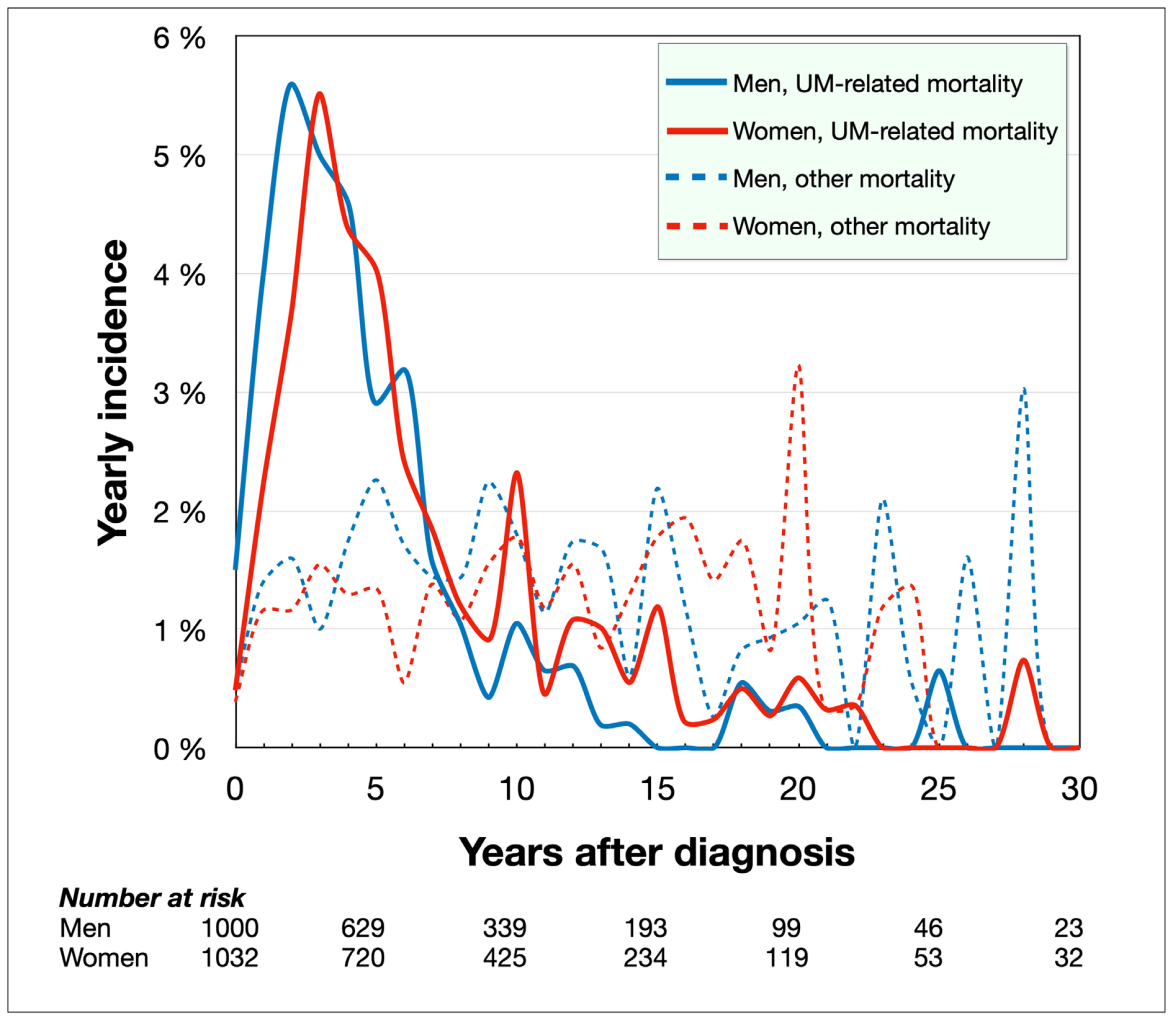
Yearly incidence of UM‐related mortality. The peak UM‐related mortality rate for women occurred slightly later than for men (5.5% of remaining population during the fourth year after diagnosis for women vs. 5.6% during the third year for men). During eight of the 10 years in the period 10–20 years after diagnosis, women had higher yearly mortality rates.

The cumulative incidence function estimate of UM‐related mortality from competing risks data 5, 10, 15, 20, 25, and 30 years after diagnosis was 20, 29, 33, 35, 36, and 36% for women and 24, 31, 32, 34, 34, and 34% for men. During the period from 0 to 30 years after diagnosis, women initially had lower incidence of UM‐related mortality, but the curves crossed 14 years after diagnosis, after which men had lower incidence of UM‐related mortality. Overall, the incidence of UM‐related mortality did not differ between men and women (Gray's test for equality *p* = 0.39, Figure 2A). Similarly, in the first decade after diagnosis, the incidence of UM‐related mortality did not differ between men and women (*p* = 0.083, Figure 2B). After the first decade however, women had significantly higher incidence of UM‐related mortality (*p* = 0.036, Figure 2C). The yearly incidence of UM‐related mortality was slightly offset so that the peak rate for women occurred slightly later than for men (5.5% of remaining population during the fourth year after diagnosis for women vs. 5.6% during the third year for men, Figure 3).

**FIGURE 4:**
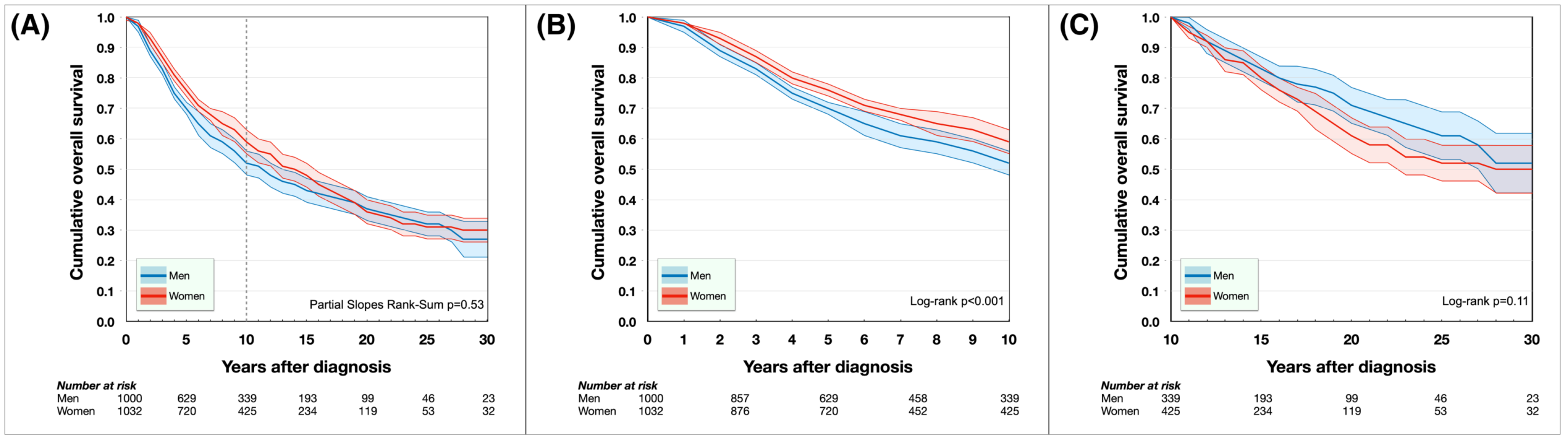
Cumulative overall survival proportions. (A) During the period from 0 to 30 years after diagnosis, there was no significant difference in overall survival between men and women (Partial Slopes Rank‐Sum *p* = 0.53). (B) Women had better overall survival in the first decade after diagnosis (*p* = 0.001). (C) In the later period however, there was no significant difference (*p* = 0.11).

The Kaplan–Meier estimate of overall survival 5, 10, 15, 20, 25, and 30 years after diagnosis was 76, 59, 48, 36, 31, and 30% for women and 70, 52, 43, 37, 32, and 27% for men. Women initially had better overall survival, but the curves crossed 18 years after diagnosis, after which men had better survival. Overall, the survival distributions did not differ (Partial Slopes Rank‐Sum *p* = 0.53, Figure 4A). Women had better overall survival in the first decade after diagnosis (Log‐rank *p* < 0.001, Figure 4B), but not thereafter (Log‐rank *p* = 0.11, Figure 4C).

### 3.5 Other causes of death

The cumulative incidence function estimate of death from other causes than uveal melanoma 5, 10, 15, 20, 25, and 30 years after diagnosis was 7, 13, 20, 29, 32, and 32% for women and 9, 17, 25, 29, 33, and 37% for men (Gray's test for equality *p* = 0.10). Metastatic UM was the leading cause of death for women during the entire period 1–30 years after diagnosis. For men, metastatic UM was the leading cause of death during the first 27 years (supplemental table).

## 4 REGRESSION ANALYSES

### 4.1 Uveal melanoma‐related death <10 years

For the early period, male sex (HR 1.2, 95% CI 1.0 to 1.4, *p* = 0.049), patient age at diagnosis (HR 1.1 per increased decade, 95% CI 1.0 to 1.2, *p* < 0.001), tumor diameter (HR 1.2 per increased millimeter, 95% CI 1.2 to 1.2, *p* < 0.001), and tumor thickness (HR 1.2 per increased millimeter, 95% CI 1.1 to 1.2, *p* < 0.001) were all significant predictors of UM‐related mortality in univariate Cox regression. Male sex did not retain its significance in multivariate analysis (HR 1.1, 95% CI 0.9 to 1.4, *p* = 0.22, Table 5A).

**TABLE 5:** Cox regression hazard for uveal melanoma‐related mortality

A, <10 years after diagnosis	B	SE	Wald	*p*	Exp(B)	95% CI lower	95% CI upper
Univariate							
Male sex	0.2	0.09	3.9	0.049	1.2	1.0	1.4
Age at diagnosis^a^	0.1	0.03	12.1	<0.001	1.1	1.0	1.2
Tumor diameter, mm^b^	0.2	0.01	230.6	<0.001	1.2	1.2	1.2
Tumor thickness, mm^b^	0.2	0.01	135.8	<0.001	1.2	1.1	1.2
Multivariate							
Male sex	0.1	0.1	1.5	0.22	1.1	0.9	1.4
Age at diagnosis^a^	0.09	0.04	7.0	0.008	1.1	1.0	1.2
Tumor diameter, mm^b^	0.2	0.01	123.3	<0.001	1.2	1.1	1.2
Tumor thickness, mm^b^	0.06	0.02	9.8	0.002	1.1	1.0	1.1

B, >10 years after diagnosis	B	SE	Wald	*p*	Exp(B)	95% CI lower	95% CI upper
Univariate							
Female sex	0.6	0.3	4.5	0.034	1.8	1.0	3.1
Age at diagnosis^a^	−0.1	0.1	1.5	0.22	0.9	0.7	1.1
Tumor diameter, mm^b^	0.07	0.04	3.2	0.072	1.1	1.0	1.1
Tumor thickness, mm^b^	0.05	0.05	1.0	0.32	1.1	1.0	1.2

^a^ Per increasing decade.

^b^ Per increasing mm.

### 4.2 Uveal melanoma‐related death ≥10 years

For the late period, female sex was the only significant predictor of UM‐related mortality in univariate Cox regression (HR 1.8, 95% CI 1.0 to 3.1, *p* = 0.034, Table 5B). As none of the other examined variates (patient age at diagnosis, tumor diameter at diagnosis or tumor thickness at diagnosis) were significant predictors, no multivariate analysis was performed.

## 5 DISCUSSION

In this study, women were demonstrated to have better overall survival in the first decade after diagnosis of UM, and significantly higher incidence of UM‐related mortality in the later period. Similarly, male and female sex were predictors of UM‐related mortality in the early and late periods in univariate analysis, respectively. Although there were no significant differences in mean age at diagnosis, tumor thickness, diameter, ciliary body involvement, primary treatment, or AJCC T‐category between men and women at baseline, men surviving ≥10 years were significantly younger.

In 2020, Dogrusöz et al. used a similar methodology as described herein, but compared prognostic factors including patient sex at an earlier point in time.^34^ In that study, male sex was associated with UM‐related death in patients that were alive 5 years after enucleation in multivariate regression with a range of covariates including patient age, tumor size, mitotic counts, tumor cell types, and chromosomes 3 and 8 status. In closer examination of their data, it appears that their cumulative incidence of UM‐related death had a comparable development with the incidence curve for women overtaking the incidence curve for men approximately 11 years after diagnosis. It may therefore be suspected that the results of our two studies would have been similar if I would have chosen the same point in time as cutoff. It should also be expected that the data analyzed by Dogrusöz et al. did not meet the proportional hazards assumption. Relatedly, in the cohorts published by Kujala et al. 2003 and Stålhammar et al. 2019, similar patterns of increasing incidences of UM‐related mortality for women relative to men 5 or 10 years after diagnosis can be discerned.^21^, ^23^ The pattern is however not apparent in the articles by Zloto et al. 2013 and Park et al. 2017.^16^, ^17^ Park et al. reported a relatively short mean follow‐up of 5.4 years. Zloto et al. described a two‐fold excess of male melanoma‐related mortality in the first 10 years after diagnosis but did not report median follow‐up for survivors or how many of their patients remained in their data after the first decade.

One possible explanation for the worse survival of women after the first decade may be inferred from the characteristics of the patients remaining in relation to baseline in the present study: Men died in greater rates in the first decade and left a group of survivors that were relatively young. Women, on the other hand, died in relatively fewer numbers in the first decade, which left a group of relatively old survivors. Age is a strong prognostic factor in UM, with a positive correlation between age and metastatic risk.^8^ This may be at least partially attributable to a lead time bias were older patients have overrepresentation of larger tumors that have grown for a longer period of time.^35^ Nevertheless, the correlation between age and poor prognosis has been retained in matched cohorts and multivariate analyses.^36^, ^37^ The correlation between patient age and metastatic risk is however not necessarily linear, why its inclusion in regression analyses might be criticized.^8^

Considering that men had a greater rate of deaths from other causes in the first decade after diagnosis, it is possible that other risk factors than age were overrepresented in this group and thereby underrepresented in the survivors. Hormonal or reproductive factors may be involved. Men and nulliparous women have an increased risk for UM metastasis and death compared with parous women.^38^, ^39^ In breast cancer, this effect has been attributed to a crossover phenomenon in which women are at increased risk of developing a tumor shortly after giving birth but at decreased risk in the long term. Presumably, this may be caused by increased immune surveillance in the wake of fetal antigen exposure during pregnancy.^40^ Such a phenomenon should not be expected in UM, as the situation was the opposite and women rather had decreased risk in the short term. Nevertheless, mounting evidence indicates that at least a subset of UM express estrogen receptors (ER) and luteinizing hormone‐releasing hormone (LHRH) receptors, and that ER expression may correlate to poor prognostic factors such as epithelioid or mixed tumor cell type, *BAP1* mutations and monosomy of chromosome 3.^41^, ^42^, ^43^ Hypothetically, increased long‐term ER or LHRH stimulation may promote tumor cells with such features and thereby increase the risk for metastasis.

This study has several limitations. First, the data were retrospective and non‐randomized, and I had limited knowledge of confounding factors. There were no baseline differences in patient age, tumor thickness, tumor diameter, or ciliary body involvement between men and women. Other than the potential dissimilarity in hormonal and reproductive factors, it is fully possible that there were differences in other established predictors. For example, gene expression class, *BAP1, SF3B1, EIF1AX*, and chromosome 3 status, presence or absence of vasculogenic mimicry, and tumor cell type are all important prognostic factors that were not accounted for herein.^44^, ^45^, ^46^, ^47^, ^48^, ^49^ Mutations in *SF3B1* have been associated with late onset of metastases at a median of 8 years after diagnosis but no sex predilection has been observed in previous studies.^50^, ^51^, ^52^ The reader should be aware that the worse survival for women in the second decade after UM diagnosis and beyond is not necessarily caused by increased presence of a poor prognostic factor in the women surviving ≥10 years. The same effect may be observed with an increased presence of a poor prognostic factor in men dying <10 years, which would leave a male population with better survival probability for the late period. A similar effect has been observed previously in terms of increasing relative survival for UM patients surviving >20 years after diagnosis, which could be the result of accumulation of risk factors in patients dying earlier.^2^, ^29^ Second, calculations of the incidence of UM‐related mortality rely on accurate classifications of the cause of death. Even though efforts had been made to reduce the number of classification errors in the national digitalized UM treatment registry by crosschecking the data against other diagnoses in the national Cancer Registry and against hospital medical records, it cannot be excluded that a proportion of deaths were misclassified. Third, the patients were sampled from one nation only and even though their survival rates were similar to several previous observations including studies from Denmark, the Netherlands, the United States, and Australia, global generalizability of the findings cannot be guaranteed.^10^, ^34^, ^53^, ^54^

In conclusion, a notion that male sex is associated with a worse prognosis in UM may be nuanced: It might indeed be true in the first decade after diagnosis. After 10 years however, female survivors are significantly older than men and have a higher incidence of UM‐related mortality. To the best of my knowledge, this is the first study to demonstrate this sex‐based difference in early and late UM‐related mortality. This can have important consequences for our current understanding of this disease. Further, the findings may be considered when examining potential sex‐related differences in prognostic factors including ER, LHRH, and other hormonal axes, immune surveillance, and lifestyle, as well as when informing patients and relatives about the disease. Future studies should aim to clarify the causal factor for the sex‐based differences, and if it can be targeted with therapeutic interventions.

## AUTHOR CONTRIBUTIONS

**Gustav Stålhammar:** Conceptualization; data curation; formal analysis; funding acquisition; investigation; methodology; project administration; resources; validation; visualization; writing – original draft.

## FUNDING INFORMATION

Support was provided to Dr. Stålhammar from: The Royal Swedish Academy of Sciences (reference ME2019‐0036); The Swedish Cancer Society (200,798 Fk); The Swedish Eye Foundation (reference 2021‐04‐28); Karolinska Institutet (reference FS‐2021‐0010); Region Stockholm (reference RS 2019‐1138). Carmen and Bertil Regnér Foundation (reference 2020–00062); The funding sources had no role in the planning or design of this study, in data collection, analysis, interpretation, or in any other aspect pertinent to the study. The author was not paid to write this article by a pharmaceutical company or other agency. The author was not precluded from any data in the study, and accepts responsibility to submit for publication.

## CONFLICT OF INTEREST

The author has no competing interests.

## Supporting information


Appendix S1:

Appendix S2:

## Data Availability

The raw data used for this study are available as a supplementary file.
