# Supplementary material for: Sex‐based differences in early and late uveal melanoma‐related mortality
Source: Cancer Med. 2022 Nov 18;12(6):6700–10. doi: 10.1002/cam4.5458 (PMC10067119; doi:10.1002/cam4.5458)
Supplement: Supplementary file 2 — Appendix S2: [file CAM4-12-6700-s001.pdf]

| Supplemental table. Incidences of uveal melanoma-related death and death from other causes. | | | | | | |
|---|---|---|---|---|---|---|
| | **Women** | | | **Men** | | |
| **Year after diagnosis** | **Cumulative incidence of melanoma-related death** | **Cumulative incidence of other death** | **Proportion melanoma-related to all deaths** | **Cumulative incidence of melanoma-related death** | **Cumulative incidence of other death** | **Proportion melanoma-related to all deaths** |
| **1** | 0.03 | 0.02 | 0.64 | 0.06 | 0.02 | 0.74 |
| **2** | 0.06 | 0.03 | 0.70 | 0.11 | 0.04 | 0.76 |
| **3** | 0.12 | 0.04 | 0.74 | 0.16 | 0.05 | 0.78 |
| **4** | 0.16 | 0.06 | 0.75 | 0.21 | 0.06 | 0.77 |
| **5** | 0.20 | 0.07 | 0.75 | 0.24 | 0.09 | 0.74 |
| **6** | 0.23 | 0.07 | 0.75 | 0.27 | 0.10 | 0.72 |
| **7** | 0.25 | 0.09 | 0.74 | 0.28 | 0.12 | 0.71 |
| **8** | 0.26 | 0.10 | 0.72 | 0.29 | 0.13 | 0.69 |
| **9** | 0.27 | 0.12 | 0.70 | 0.30 | 0.15 | 0.66 |
| **10** | 0.29 | 0.13 | 0.69 | 0.31 | 0.17 | 0.64 |
| **11** | 0.30 | 0.14 | 0.67 | 0.32 | 0.18 | 0.63 |
| **12** | 0.31 | 0.16 | 0.66 | 0.32 | 0.20 | 0.62 |
| **13** | 0.32 | 0.17 | 0.65 | 0.32 | 0.22 | 0.60 |
| **14** | 0.32 | 0.18 | 0.64 | 0.33 | 0.22 | 0.59 |
| **15** | 0.33 | 0.20 | 0.63 | 0.33 | 0.25 | 0.57 |
| **16** | 0.34 | 0.22 | 0.61 | 0.33 | 0.26 | 0.56 |

| | | | | | | |
|---|---|---|---|---|---|---|
| **17** | 0.34 | 0.23 | 0.59 | 0.33 | 0.26 | 0.56 |
| **18** | 0.34 | 0.25 | 0.58 | 0.33 | 0.27 | 0.55 |
| **19** | 0.35 | 0.26 | 0.57 | 0.34 | 0.28 | 0.55 |
| **20** | 0.35 | 0.29 | 0.55 | 0.34 | 0.29 | 0.54 |
| **21** | 0.35 | 0.29 | 0.55 | 0.34 | 0.30 | 0.53 |
| **22** | 0.36 | 0.30 | 0.55 | 0.34 | 0.30 | 0.53 |
| **23** | 0.36 | 0.31 | 0.54 | 0.34 | 0.32 | 0.51 |
| **24** | 0.36 | 0.32 | 0.53 | 0.34 | 0.33 | 0.51 |
| **25** | 0.36 | 0.32 | 0.53 | 0.35 | 0.33 | 0.51 |
| **26** | 0.36 | 0.32 | 0.53 | 0.35 | 0.34 | 0.50 |
| **27** | 0.36 | 0.32 | 0.53 | 0.35 | 0.34 | 0.50 |
| **28** | 0.37 | 0.32 | 0.53 | 0.35 | 0.37 | 0.48 |
| **29** | 0.37 | 0.32 | 0.53 | 0.35 | 0.37 | 0.48 |
| **30** | 0.37 | 0.32 | 0.53 | 0.35 | 0.37 | 0.48 |
